# Development, validation and regulatory acceptance of improved purification and simplified quality control of [^13^N] Ammonia

**DOI:** 10.1186/s41181-020-00097-7

**Published:** 2020-05-13

**Authors:** Daniel L. Yokell, Peter A. Rice, Ramesh Neelamegam, Georges El Fakhri

**Affiliations:** 1 Department of Radiology, Gordon Center for Medical Imaging, Massachusetts General Hospital, 55 Fruit Street, Edwards 019B, Boston, MA 02114 USA; 2 Department of Radiology, Harvard Medical School, Boston, MA USA

**Keywords:** [^13^N]Ammonia, [^13^N]NH_3_, Cardiac PET, Automated radiochemistry, Regulatory, cGMP PET drug manufacturing, PET radiopharmaceutical quality control

## Abstract

**Background:**

[^13^N]Ammonia is a cyclotron produced myocardial perfusion imaging agent. With the development of high-yielding [^13^N]ammonia cyclotron targets using a solution of 5 mM ethanol in water, there was a need to develop and validate an automated purification and formulation system for [^13^N]ammonia to be in a physiological compatible formulation of 0.9% sodium chloride since there is no widely available commercial system at this time. Due to its short half-life of 10 min, FDA and USP regulations allow [^13^N]ammonia to be tested in quality control (QC) sub-batches with limited quality control testing performed on the sub-batches for patient use. The current EP and the original USP method for the determination of the radiochemical purity and identity of [^13^N]ammonia depended on an HPLC method using a conductivity detector and a solvent free of other salts. This HPLC method created issues in a modern cGMP high volume PET manufacturing facility where the HPLC is used with salt containing mobile phase buffers for quality control analysis of other PET radiopharmaceuticals. Flushing of the HPLC system of residual salt buffers which may interfere with the [^13^N]ammonia assay can take several hours of instrument time. Since there are no mass limits on [^13^N]ammonia, a simplified TLC assay to determine radiochemical identity and purity could be developed to simplify and streamline QC.

**Results:**

We have developed and validated a streamlined automated synthesis for [^13^N]ammonia which provides the drug product in 8 mL of 0.9% sodium chloride for injection. A novel radio-TLC method was developed and validated to demonstrate feasibility to quantitate [^13^N]ammonia and separate it from all known radiochemical impurities.

**Conclusions:**

The process for automated synthesis of [^13^N]ammonia simplifies and automates the purification and formulation of [^13^N]ammonia in a cGMP compliant manner needed for high-throughput manufacture of [^13^N]ammonia. The novel radio-TLC method has simplified [^13^N]ammonia quality control (QC) and now enables it to be tested using the same QC equipment as [^18^F]fludeoxyglucose (FDA/USP recognized name for 2-[^18^F]fluoro-2-deoxy-D-glucose). Both the streamlined automated synthesis of [^13^N]ammonia and the novel radio-TLC method have been accepted and approved by the US Food and Drug Administration (FDA) for the cGMP manufacture of [^13^N]ammonia.

## Background

[^13^N]Ammonia is a myocardial perfusion imaging agent, which is approved by US Food and Drug Administration (FDA) for diagnostic Positron Emission Tomography (PET) imaging of the myocardium under rest or pharmacologic stress conditions to evaluate myocardial perfusion in patients with suspected or existing coronary artery disease.(Dilsizian et al. 2016) In the United States, the only other FDA approved alternative PET myocardial perfusion agent is [^82^Rb]rubidium chloride, which requires a generator system by the patient’s side due to the short half-life. With advances in cyclotron capabilities and targetry associated with in-target [^13^N]ammonia production, in excess of 37 GBq (1 Ci) of [^13^N]ammonia can be produced per batch which makes it feasible with the 10-min half-life to inject and image more than one patient per batch and even transport it short distances from the cyclotron. Due to the expanding infrastructure globally of cyclotron and PET cameras, there has been rapidly increasing interest in [^13^N]ammonia for PET myocardial perfusion imaging outside of the US.(Underwood et al. 2014)

The [^13^N]ammonia in-target production method produces [^13^N]ammonia in water with 5 mM ethanol.(Wieland et al. 1991) This formulation vehicle while acceptable, is less than ideal than a physiological compatible solution like 0.9% sodium chloride. Additionally, the [^13^N]ammonia in water may contain trace long lived radionuclidic impurities from the target body and/or target windows depending on the cyclotron target design. Most of the existing methods described for purification and formulation of [^13^N]ammonia are either manual loading and elution of solid phase extraction cartridges (SPEs) or complicated dedicated [^13^N]ammonia systems, both of which are challenging to validate in a cGMP manufacturing environment.(Frank et al. 2019; Pieper et al. 2019; Kumar et al. 2009) We set out to design a simple method which could be adapted and validated on several different commercial available platforms, including cassette based systems for easy scale up for high-volume [^13^N]ammonia production at a busy PET center.

The original United States Pharmacopeia (USP)([^13^N]Ammonia Monograph n.d.-a) and European Pharmacopeia (EP)([^13^N]Ammonia Monograph n.d.-b) methods for radiochemical purity and identity determination of [^13^N]ammonia currently require the use of an HPLC system configured with a conductivity detector. The current compendial EP and the original USP HPLC conductivity methods are resource intensive, requiring lengthy mobile phase preparation and system suitability determination, while occupying valuable instrument time. Additionally, these detectors are only required for anion or cation chromatography, which is an expensive investment for a PET center to use only for [^13^N]ammonia, sodium [^18^F] fluoride and other investigational cation/anion radiopharmaceuticals. In our experience, use of a conductivity detector on an HPLC also used for traditional reverse phase chromatography can create system suitability and baseline noise issues in the cation analysis if proper care is not taken to properly flush the system lines of salt buffers used in reverse phase analysis. System suitability failures can lead to extensive delays in the busy production schedule of PET radiopharmaceuticals.

In this paper, we describe the development, validation and regulatory acceptance by FDA and USP of an ideal alternative method that would rapidly and reproducibly determine the radiochemical purity and radiochemical identity of [^13^N]ammonia, while requiring less time to complete and fewer resources to maintain than compendial methods. A TLC method for determining radiochemical purity and identity eliminates the need to use HPLC for [^13^N]ammonia quality control. This is highly desirable in a PET production facility that produces multiple radiopharmaceuticals per day.

## Materials and methods

### Chemicals and reagents

All chemicals and reagents were obtained from commercial vendors and used without further purification.

### Automated purification and formulation of [^13^N]Ammonia

**Fig. 1:**
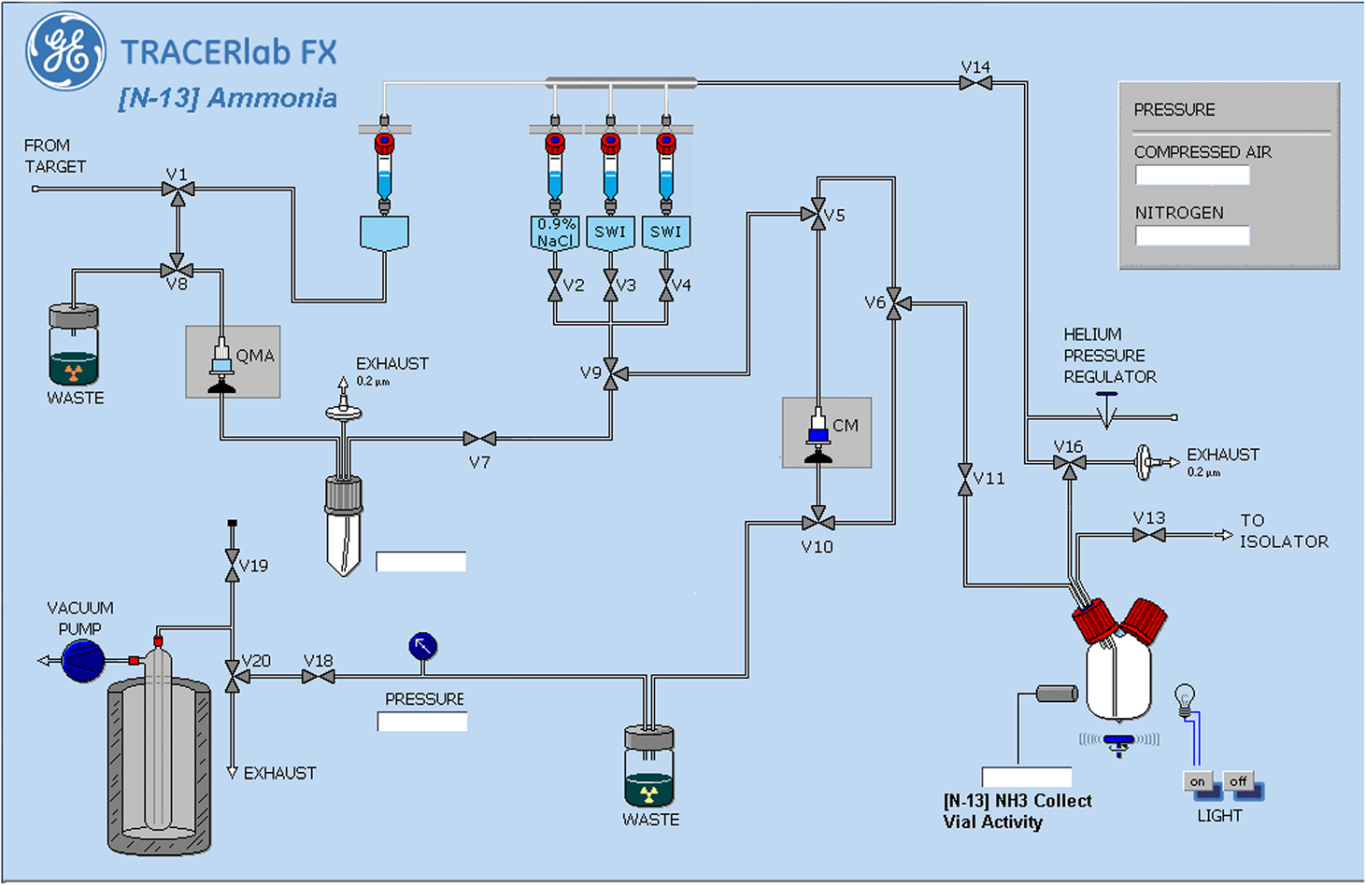
[^13^N]Ammonia Purification and Formulation Module Graphic User Interface

[^13^N]Ammonia is produced on a modified GE Tracerlab FXFDG synthesis module which was replumbed for the purification and formulation of [^13^N]ammonia. The graphic user interface is shown in Fig. 1 and the purification and formulation steps are further detailed below. The QMA chloride and CM cartridges are prepared day of use by washing with 10 mL of sterile water for injection, USP and then capping the cartridges with sterile male/female luer caps. Prior to the first sub-batch of the day, the synthesis box vacuum is tested and the system fluid paths are washed with sterile water for injection, USP. The cyclotron [^13^N]ammonia target prior to the day’s use is primed with fresh 5 mM ethanol solution.

1. [^13^N]Ammonia is produced on-site with a GE PETrace 860 cyclotron using the method(Wieland et al. 1991) of irradiation of 5 mM ethanol in water in-target synthesis method using a GE niobium body target with HAVAR/Niobium double target window or GE silver body target with HAVAR window (GE Medical Systems, Uppsala, Sweden). The target solution is pressurized to approximately 0.62 MPa (90 psi) for the silver body target or to approximately 1.31 MPa (190 psi) for the niobium body target with helium gas. The target is bombarded with 16.5 MeV protons using a beam current ranging from 15 to 50 μA using bombardment times between 5 and 30 min. For the [^13^N]ammonia batches cited in Tables 1, 50 μA, 30 min bombardments were used.
2. [^13^N]Ammonia is transferred from the cyclotron target via 0.45 MPa (65 psi) helium overpressure to the automated synthesis unit and through an in-line anion exchange column (Waters, QMA Chloride) to remove any anionic impurities, such as [^18^F]fluoride.
3. Using vacuum, the [^13^N]ammonia in water is trapped on a cation exchange column (Waters, Accell CM) to quantitatively trap [^13^N]ammonia.
4. The [^13^N]ammonia is released from the cation exchange column using 8 mL of 0.9% sodium chloride for injection, USP.
5. The formulated [^13^N]ammonia in 0.9% sodium chloride for injection is then transferred through a 1/16″ PFA line via 0.1 MPa (14.5 psi) nitrogen overpressure to a ISO Class 5 isolator for sterile filtration through a vented 0.22 μ polyethersulfone (PES) membrane filter (B Braun) into a vented 30 mL sterile empty vial (ALK OKC Allergy Labs, Hollister Stier or Huayi Isotopes).

**Table 1:** Summary Table of [^13^N]Ammonia Injection Stability Test Average Results from Five Batches compared to the FDA/USP Test Specifications

Quality control specification	Acceptance criteria	Average result
Activity at End of Synthesis	1.11–76.96 GBq @ EOS 30 to 2080 mCi @EOS	10.18 ± 0.22 GBq 572 ± 60 mCi
Product Volume	8 mL ± 20%	7.61 ± 0.2 mL
pH Determination	4.5–7.5	5
Visual Inspection	Clear, colorless solution. Absent of foreign matter. Product vial is intact.	Pass
Radionuclidic Identity	Principal photopeaks are found at 0.511 MeV, 1.02 MeV and Compton scatter	Pass
Half-life Determination (minutes)	The measured half-life is between 9.5–10.5 min	9.99 ± 0.1 min
Radiochemical Identity	Rf of resazurin = 0.43–0.63	0.5 ± 0.05
Radiochemical Purity	NLT 95.0% Ammonia N 13 via TLC	98.61 ± 0.4%
Residual Solvent Assay	Ethanol NMT 3.1 mg/mL	<LLOD to 0.5 mg/mL
Sterile Filter Integrity	≥ manufacturer specification of 46 psi	> 46 psi
Bacterial Endotoxin Testing (EU/mL)	NMT 10.9 EU/mL	< 5 EU/mL
Sterility	Sterile	Sterile
Long Lived Radionuclidic Purity	< 0.5% at time of expiry	< 0.001% (less than lower limit of detection)

*Abbreviations*: *EOS* end of synthesis, *EU* endotoxin units, *LLOD* lower limit of detection, *NLT* not less than, *NMT* not more than

The total time of the purification, formulation and sterile filtration of the [^13^N]ammonia takes approximately 5 min.

Post-formulation in between sub-batches, the fluid pathways in the system unit and the transfer line are washed with sterile water for injection, USP and blown to dryness with nitrogen gas.

### Quality control of [^13^N]Ammonia

The quality control of [^13^N]ammonia was performed to ensure the PET drug product met the specifications in Table 1 to satisfy FDA and USP regulatory requirements. Due to the production of [^13^N]ammonia via in-target 5 mM ethanol solution, the EP/USP test for residual aluminum was not required. The TLC method was validated against the major potential radiochemical impurities, [^13^N]NOx and [^18^F]fluoride as detailed in Table 2 and cross-validated against the compendial HPLC method.

**Table 2:** [^13^N]Ammonia TLC method validation results

[^**13**^N]Ammonia	% [^13^N]Ammonia integrated	Peak Start (mm)	Peak End (mm)	Peak centroid (mm)	Rf Value	Resazurin Standard Range (mm)*
[^13^N]Ammonia – TLC Strip #1	99	20.3	81.8	50.8	0.846	0–40
[^13^N]Ammonia – TLC Strip #2	98.96	17.7	79.2	51.7	0.862	0–40
[^13^N]Ammonia – TLC Strip #3	99.06	17.7	80.1	51.2	0.853	0–45
***Mean***	***99.01%***				***0.854***	
**[**^**18**^**F]Fluoride**	%[^18^F]Fluoride integrated	Peak Start (mm)	Peak End (mm)	Peak centroid (mm)	Rf Value	Resazurin Standard Range (mm)*
[^18^F]Fluoride – TLC Strip #1	99.5	− 17.3	30.5	7.1	0.119	0–41
[^18^F]Fluoride – TLC Strip #2	99.78	−16.5	25.4	4.3	0.072	0–44
[^18^F]Fluoride – TLC Strip #3	99.77	−15.6	30.5	5.7	0.095	0–44
***Mean***	***99.68***				***0.095***	
**[**^**13**^**N]NOx**	% [^13^N]NOx integrated	Peak Start (mm)	Peak End (mm)	Peak centroid (mm)	Rf Value	Resazurin Range (mm)*
[^13^N]NOx – TLC Strip #1	98.34	−12.4	14.4	0.1	0.00	0–40
[^13^N]NOx – TLC Strip #2	97.94	−13.2	13.5	−0.8	−0.01	0–41
[^13^N]NOx – TLC Strip #3	98.21	−14.1	14.4	− 0.6	−0.01	0–40
***Mean***	***98.16***				***−0.01***	
**[**^**18**^**F]Fluoride / [**^**13**^**N]Ammonia Mixed Solution (Co-spot)**	% integrated	Peak Start (mm)	Peak End (mm)	Peak centroid (mm)	Rf Value	Resazurin Range (mm)*
[^18^F]Fluoride (4% impurity)	3.6%	−12.1	24.75	18	0.3	0–44
[^13^N]Ammonia (96% purity)	96.4%	31.5	80.1	49.5	0.82	
***Total***	***100%***					
***Accuracy of [***^***18***^***F]Fluoride measurement***	***90% of expected value***					
***Accuracy of [***^***13***^***N]Ammonia measurement***	***100.4% of expected value***					

*A valid system suitability result requires the front of the resazurin spot to be 34–50 mm

A thin layer chromatographic system was developed that uses a diethylaminoethyl cellulose (DEAE-C) stationary phase. The DEAE-C stationary phase of the chromatography system was chosen due to its ability to attract anionic species. The DEAE-C strip (J.T. Baker) is 1.5 cm × 8 cm and the mobile phase is composed of methanol: water 75:25. The Rf of [^13^N]ammonia is 0.7–0.9 and the major impurities of [^13^N]NOx and [^18^F]fluoride are retained at the origin (Rf = 0). A 0.5 μL spot of [^13^N]ammonia is applied to the left hand side of the origin of the TLC strip via pipette and a 0.5 μL spot of 100 mg/mL ammonium chloride reference standard, USP is applied to the right hand side of the origin of the TLC strip. Both spots were allowed to dry prior to the TLC strip development in the mobile phase of methanol: water 75:25. The time for TLC strip development is approximately 8–10 min in a tightly sealed development chamber. Following development of the TLC strip, the strip was allowed to dry and was then counted using an AR-2000 radio-TLC plate reader (Eckert and Ziegler). Originally, the radiochemical identity was confirmed with 100 mg/mL ammonium chloride USP reference standard which was visualized with a combination of spray the TLC strip with iodoplatinate reagent, which was allowed to develop for 10 min followed by placement in an iodine chamber for approximately 10 min to help highlight the ammonium chloride spot. The ammonium chloride spot appears as an orange brown spot against a light maroon background (Fig. 3a).

The radio-TLC method has since been simplified with the use of resazurin (Millipore Sigma), a visible dye which is used as a marker of system suitability eliminating the need to use ammonium chloride and the complicated development process of iodoplatinate reagent and iodine chamber to visualize the standard. Additionally, the visible dye aids the operator by providing a visual pink-purple streak indicating proper TLC development. A 0.5 μL spot of [^13^N]ammonia is applied to the left hand side of the origin of the TLC strip via pipette and a 0.5 μL spot of resazurin dye (1 mg/mL) is applied to the right hand side of the origin of the TLC strip. Both spots were allowed to dry prior to the TLC strip development in the mobile phase of methanol: water 75:25. The other components of the TLC assay remain the same as described above. The Rf of resazurin is 0.43–0.63 and an example TLC strip is shown in Fig. 3b. Table 2 below contains the validation data of the [^13^N]ammonia TLC assay using resazurin as a system suitability marker. For system suitability purposes, we require the front of the resazurin peak to be 34–50 mm.

Complete quality control testing and the [^13^N]ammonia test specifications for five high activity stability batches is described in Table 1. For routine clinical production, a quality control (QC) sub-batch is performed prior to manufacturing any sub-batches for patient use. The routine clinical QC sub-batch QC process takes approximately 25 min. All of the QC tests are detailed below which are performed on the quality control sub-batch, except for the periodic quality indicating tests (PQIT), which are performed at their defined testing periods. For the patient sub-batches of [^13^N]ammonia, partial quality control testing is performed, which is composed of final product vial visual inspection, product assay, and sterile filter integrity test.

Sterile filter integrity is performed using a manual bubble point test using a variable pressure gas source with a calibrated pressure gauge according to filter manufacturing directions. Visual inspection of the final product vial is performed by a qualified operator observing the vial through the lead glass window of the dispensing hot cell to verify vial integrity as well as to ensure the solution is clear and particulate free. The pH testing was performed using two pH strips (0–6, 2–9, EMD Millipore) and comparing the result to pH strips spotted with the closest US NIST traceable pH reference standards. Radio-TLC is performed to determine radiochemical purity and identity as described above using the radio-TLC method. Sterility testing is performed within 30 h of end of synthesis of the QC sub-batch using a validated modification of USP < 71> direct inoculation method of 0.1–0.3 mL of [^13^N]ammonia into TSB and FTM hungate 10 mL sterility tubes. Bacterial Endotoxin testing is performed using the Endosafe Nexgen PTS system (Charles River). Residual solvent testing for ethanol content (ICH Class III solvent), is a periodic quality indicating test (PQIT) performed at least quarterly using a GC (Agilent 7890) with direct split injection (15:1) onto a USP G16 wax column (Agilent DB Wax ETR, 30 m × 0.25 mm × 0.5 μm), using hydrogen as a carrier gas (1.3 mL/min) and FID detector. GC oven at time of injection is 40 °C, and then ramps 40 °C/min to 110 °C, where it holds for 1 min (3.25 min run time). Ethanol elutes at approximately 2.7 min. Radionuclidic identity is performed as a PQIT at least annually using a high purity germanium detector system with Genie software (Mirion) which automatically detects and identifies the 511 keV, 1022 keV, Compton scatter photopeaks as well as any unknown photopeaks. Radionuclidic purity is an annual PQIT which is performed using the high purity germanium detector system using a two-hour count to be able to quantitate 3.7 Bq (100pCi). The Genie software automatically calculates the amount of the known radionuclidic impurities feasible from the target body and target windows as well as flags any identified unknown photopeaks for further analysis and identification.

### Synthesis of [^13^N]Ammonia major radiochemical impurities, [^13^N]NOx and [^18^F]fluoride

[^13^N]NOx was produced by cyclotron bombardment of high purity water using identical irradiation conditions as described above for [^13^N]ammonia. [^13^N]NOx was used for the methods validation without further purification. [^18^F]Fluoride was produced by cyclotron bombardment via (p,n) reaction of ≥98% enriched [^18^O]water (Rotem or Taiyo Nippon Sanso) using the GE Niobium [^18^F]fluoride target with variable beam currents up to 65 μA. [^18^F]Fluoride was used for the methods validation after allowing for decay of [^13^N]-species.

## Results

**Fig. 2:**
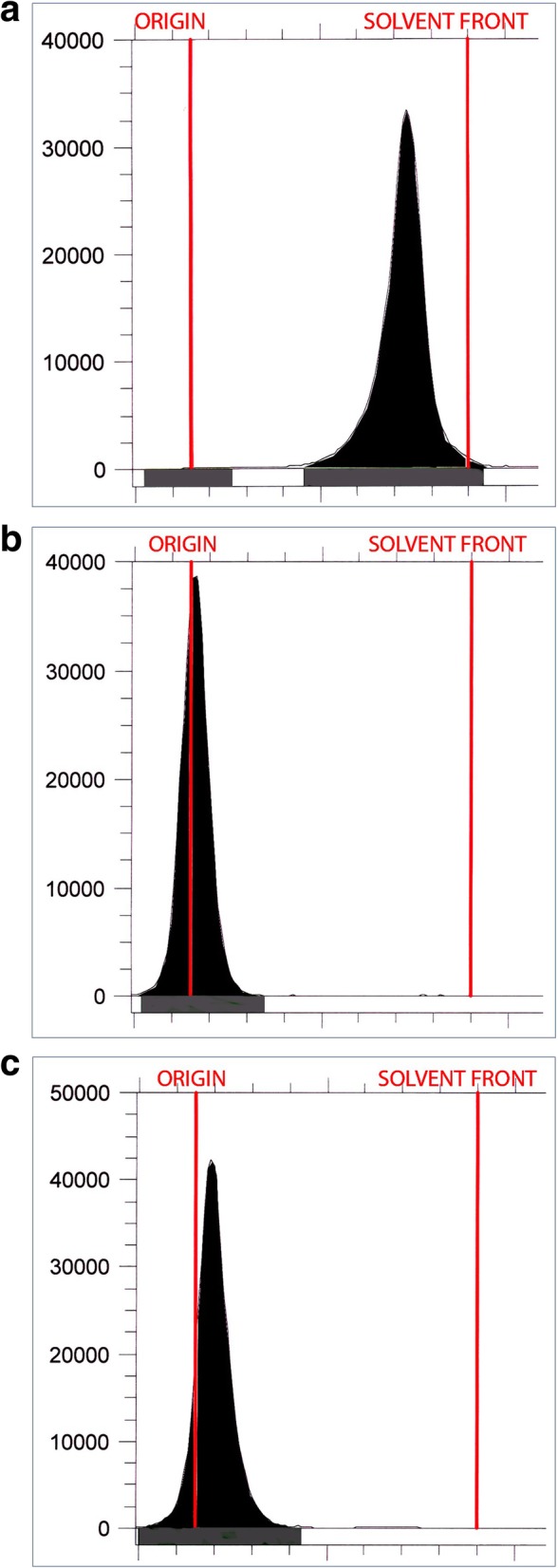
[^13^N]Ammonia Radio-TLC Chromatograms of [^13^N]ammonia (**a**), [^13^N]NOx (**b**), and [^18^F]fluoride (**c**)

**Fig. 3:**
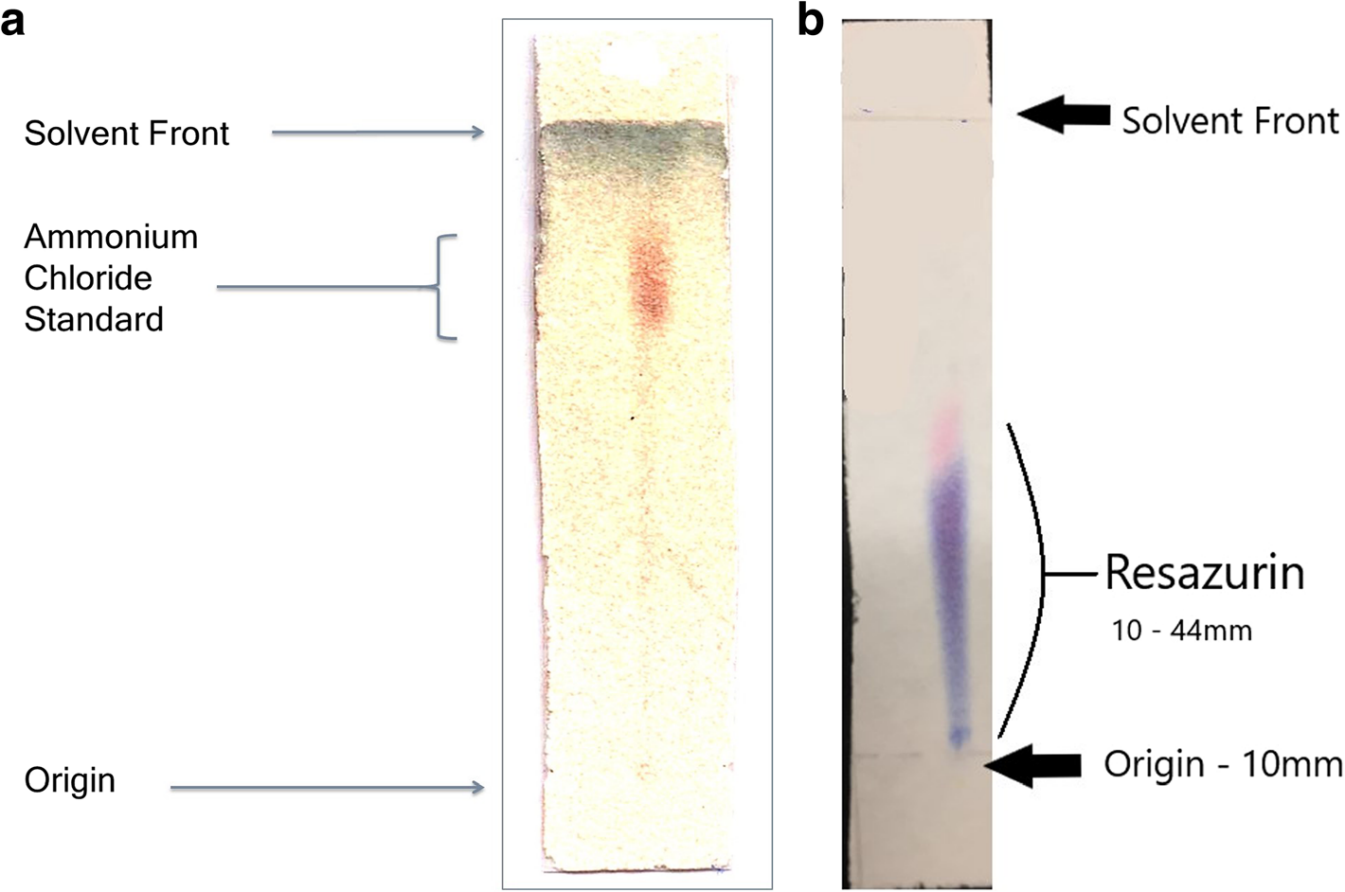
Representative [^13^N]Ammonia TLC strips showing development with ammonium chloride standard (**a**) and with resazurin (**b**)

[^13^N]Ammonia was synthesized as described above. A summary of the 2018–2019 annual validation stability studies is detailed in Table 1 with the FDA approved [^13^N]ammonia product specifications and the average results for the five batches. All of the batches met the FDA/USP product specifications. Long-lived radionuclidic analysis revealed no detectable long-lived radionuclidic impurities from the ammonia cyclotron target body or window. The validation of the radio-TLC method with resazurin as a system suitability indicator for a valid test is detailed in Table 2. We demonstrated that [^13^N]ammonia can be adequately separated from the known impurities, [^18^F]fluoride and [^13^N]nitrous oxide (NOx). The two major impurities are retained on the radio-TLC DEAE-C TLC strip at the origin while [13 N]ammonia migrates to the solvent front. [^13^N]Ammonia and [^18^F]fluoride were mixed and co-spotted on the TLC strip and counted post development. At time of measurement, the calculated percentages with within 10% of the measured percentages for [^18^F]fluoride and [^13^N]ammonia. Table 2 also contains the Rf data on the resazurin dye to validate it’s use as marker for system suitability replacing the ammonium chloride reference standard. Figure 2 shows representative radio-TLC chromatograms of [^13^N]ammonia, [^18^F]fluoride and [^13^N]NOx. Figure 3 show representative radio-TLC strips developed with the ammonium chloride standard visualized with iodoplatinate reagent/iodine vapor and resazurin indicating reagent.

## Discussion

The automated purification and formulation simplifies the operation of [^13^N]ammonia produced via the in-target production method and eliminates the need for operators to turn stopcocks or valves to purify and isolate [^13^N]ammonia for formulation. The purification process quantitatively removed all known radiochemical and radionuclidic impurities from the manufacturing process. The known radiochemical impurities which can be theoretically made in the [^13^N]ammonia cyclotron irradiation include [^18^F]fluoride, [^15^O]water and [^13^N]NO_2_^−^ / [^13^N]NO_3_^−^ (NOx). [^18^F]fluoride is quantitatively trapped on the QMA cartridge, which was documented on parts count via radioactive decay analysis. [^15^O]water is not trapped on any of the SPEs and is sent to waste. [^13^N]NOx species are quantitatively removed by the QMA cartridge. The long-lived radionuclidic impurities produced from the silver body cyclotron target with HAVAR window were identified as trapped on the QMA and CM cartridges upon further analysis. No long-lived radionuclidic impurities were identified as being carried into the manufacturing process from the niobium body cyclotron target with HAVAR/niobium double window, as niobium is the inner window.

The purification and formulation method described can be adapted with little to no modifications for use on a variety of synthesis platforms, including cassette-based systems such as the Ora Neptis, Trasis All-In-One, and GE FASTlab and MX systems. The method for cassette can be further modified to have a number of purification cartridges on the cassette to one cassette could be used for multiple runs without opening the hot cell.

The radio-TLC method whose development is described in this publication allows for [^13^N]ammonia to be simplified and eliminates the need to have a dedicated ion chromatography HPLC or add-on conductivity detector for a HPLC system. With the adoption of the novel TLC method, a PET manufacturing site can perform [^13^N]ammonia quality control using the same equipment required for [^18^F]fludeoxyglucose. The method has been accepted and adopted by the USP as the new standard for radiochemical identity and purity of [^13^N]ammonia with its publication in USP/NF 42–37 in 2019.([^13^N]Ammonia Monograph n.d.-c) We describe how the radio-TLC method has been further simplified and improved with the replacement of the ammonium chloride standard with the visible dye resazurin. The use the visible resazurin dye streamlines the radio-TLC test method as it now allows the QC operator to see at time of strip development if the TLC test is valid, saving valuable time with a short-lived isotope. Additionally, it eliminates the need to use iodoplatinate spray reagent and an iodine vapor chamber to visualize the ammonium chloride spot which can be cumbersome for an operator to perform reproducibly. The visualization with iodoplatinate reagent and iodine vapor chamber was identified as difficult to reproduce through email reports to the authors from other PET sites.

We found that the radio-TLC analysis was best performed on a proportional counting system, like the AR-2000, as the entire TLC strip is able to counted simultaneously, as well as exhibit excellent sensitivity and resolution at low activity levels. We found systems which scan the radio-TLC strip using a fixed or mobile NaI or similar detector had unacceptably high noise and peak resolution due to low counting activity, as well as scatter and decay during counting of the strip due to the 10-min half-life of [^13^N]ammonia. Alternative counting methods using cut-strip method in a well counter were not performed in our laboratory, but could theoretically be validated using the methods outlined in this paper.

Both the automated purification and formulation method for [^13^N]ammonia produced via the in-target production method and the novel radio-TLC radiochemical purity test method have been accepted by the US FDA, including the updated radio-TLC method with resazurin as a system suitability indicator for radiochemical purity. Additionally, the radio-TLC radiochemical purity method has been adopted by the USP and has replaced the radio-HPLC method which was difficult and time consuming to perform.

## Conclusion

The automated synthesis method for [^13^N]ammonia and the radio-TLC quality control assay have been thoroughly validated and are ready to support the wider use of [^13^N]ammonia globally for cardiac PET applications. The improved radio-TLC assay described in this work is a simplification of the method described in the USP monograph which improves the utility and ease of use of this assay in routine [^13^N]ammonia quality control in a cGMP environment.

## Data Availability

All data generated or analyzed during this study are included in this published article.

## Abbreviations

cGMP: current good manufacturing practices
DEAE-C: Diethylaminoethyl cellulose
EP: European Pharmacopeia
FDA: United States Food and Drug Administration
FDG: F-18 Fludeoxyglucose, 2-[^18^F]fluoro-2-deoxy-D-glucose
FTM: Fluid thioglycolate medium
HAVAR: UNS R30005, alloy of cobalt
NOx: Nitrous oxide
NIST: US National Institute of Standards and Technology
PES: Polyethersulfone
PET: Position emission tomography
PFA: Perfluoroalkoxy fluoropolymer plastic
QC: Quality control
Rf: Retention factor
TLC: Thin layer chromatography
TSB: Trypticase soy broth
USP: United States Pharmacopeia

## References

[CR1] [^13^N]Ammonia Monograph. (n.d.-a) US Pharmacopeia, Rockville. USP-NF 41–36. page 2955.

[CR2] [^13^N]Ammonia Monograph. (n.d.-b) European Pharmacopeia Ph. Eu 10, 965.

[CR3] [^13^N]Ammonia Monograph. (n.d.-c) US Pharmacopeia, Rockville. USP-NF 42–37. page 3150.

[CR4] Dilsizian V, Bacharach SL, Beanlands RS (2016). ASNC imaging guidelines/SNMMI procedure standard for positron emission tomography (PET) nuclear cardiology procedures. J Nucl Cardiol.

[CR5] Frank C, Winter G, Rensei F (2019). EJNMMI Radiopharm Chem.

[CR6] Kumar R, Singh H, Jacob M (2009). Hell J Nucl Med.

[CR7] Pieper J, Patel VN, Escolero S, et al. J Nucl Cardiol. 2019; 10.1007/s12350-019-01886-7.

[CR8] Underwood SR (2014). Eur Heart J Cardiovasc Imaging.

[CR9] Wieland B, Bida G, Padgett H (1991). Appl Radiat Isot.

